# Bottom-up driven involuntary attention modulates auditory signal in noise processing

**DOI:** 10.1186/1471-2202-11-156

**Published:** 2010-12-30

**Authors:** Lothar Lagemann, Hidehiko Okamoto, Henning Teismann, Christo Pantev

**Affiliations:** 1 Institute for Biomagnetism and Biosignalanalysis, University of Münster, Malmedyweg 15, 48149 Münster, Germany; 2 Department of Integrative Physiology, National Institute for Physiological Sciences, Okazaki, Japan

## Abstract

**Background:**

Auditory evoked responses can be modulated by both the sequencing and the signal-to-noise ratio of auditory stimuli. Constant sequencing as well as intense masking sounds basically lead to N1m response amplitude reduction. However, the interaction between these two factors has not been investigated so far. Here, we presented subjects tone stimuli of different frequencies, which were either concatenated in blocks of constant frequency or in blocks of randomly changing frequencies. The tones were presented either in silence or together with broad-band noises of varying levels.

**Results:**

In silence, tones presented with random sequencing elicited a larger N1m response than tones presented with constant sequencing. With increasing noise level, this difference decreased and even vanished in the condition where noise intensity exceeded the tone intensity by 10 dB. Furthermore, under noisy conditions, the N1m latency was shorter in the constant sequencing condition compared to the random sequencing condition.

**Conclusions:**

Besides the well-known neural habituation mechanisms, bottom-up driven attention plays an important role during auditory processing in noisy environments. This bottom-up driven attention would allow us to track a certain auditory signal in noisy situations without voluntarily paying attention to the auditory modality.

## Background

The ability of humans to disentangle and to perceptually isolate a single sound from multiple simultaneously present irrelevant sounds ("noise") is vitally important. From an evolutionary perspective, this ability might have helped noticing and spotting predators sneaking up in the midst of wind rustling in the trees or against a background of heavy rain. Of course it also plays an important role in today's everyday life (e.g., to be warned of an approaching vehicle in a diffuse traffic noise setting). The segregational and integrational mechanisms at work during this auditory scene analysis [1] are based upon the physical features of the sounds (such as spectrum, intensity, phase, etc.) coming from distinct or identical sources.

Besides these features neural activity in the auditory cortex is also affected by the sequencing of sounds in time. Repeated applications of a stimulus can decrease the corresponding neural activity [2]. This phenomenon, called habituation, appears on several time scales [3,4], at several stages of the auditory system [5,6], and is reflected in auditory evoked potentials [7-9] and auditory evoked fields [10]. Budd and colleagues [9] note that N1 response [11] decrements can stem from different mechanisms, habituation as well as refractoriness. A further term used to describe the phenomenon of neural response decline during sensory stimulation is adaptation [4,12]. This term refers to a different possible neural mechanism; however, it would as well result in a decrement of neural activity after repetition of identical stimuli.

In a recent experiment, Okamoto et al. [13], [14] presented subjects with tonal stimuli of different frequencies embedded in band-eliminated noises and measured their magnetoencephalographic (MEG) activity. They found that the tonal stimuli of randomly changing frequencies elicited a smaller N1m response (the magnetic counterpart of the electrical N1 response) than stimuli of identical frequency. At first glance, the relatively stronger neural activity elicited by identical auditory stimuli was contrary to what one would have expected considering the habituation effect. The authors hypothesized that in a noisy environment bottom-up and/or top-down driven attention could have compensated for the habituation effect. However, the use of the band-eliminated noises entailed that the tonal stimuli were centred in a silent band within background noise. This led to a different pre-stimulus noise exposure depending on the sequencing condition. In the constant sequencing condition the band-eliminated noises never masked the neural population in the frequency range of the tone stimuli. In the random sequencing condition on the other hand, the frequency region of the respective tonal stimulus had always been covered shortly beforehand by the noise from the preceding stimulation. This might have caused different short- and long-term habituation effects on the neural groups corresponding to the frequencies within and outside of the eliminated bands. Additionally, the authors always used noises of identical intensity. Therefore, the question still remains whether in noisy environments of different level under non-attentive listening conditions the neural activity in human auditory cortex can be enhanced by constant sound signal sequencing. With the present study, we attempted to address this general question and to investigate interactive effects of stimulus sequencing and masking noise level in the auditory cortex. We presented subjects amplitude modulated pure-tone stimuli differing in sequencing (constant vs. random); furthermore, the level of simultaneously presented masking noise (no noise vs. medium noise level vs. high noise level) was varied. We adopted amplitude modulated tones in order to elicit both, the auditory steady state as well as the N1m response [15]. To cause similar masking and habituation effects on the different frequency test tone stimuli, we used broad-band noise.

## Methods

### Subjects

17 healthy subjects (10 females, age range 21-30 years old) participated in the study. All subjects were distinctly right handed (assessed with the Edinburgh Handedness Inventory [16]) and had normal hearing. All subjects were fully informed about the study and gave written informed consent for their participation in accordance with procedures approved by the Ethics Commission of the Medical Faculty, University of Münster. The study thus conforms to The Code of Ethics of the World Medical Association (Declaration of Helsinki).

### Stimuli and experimental design

**Figure 1:**
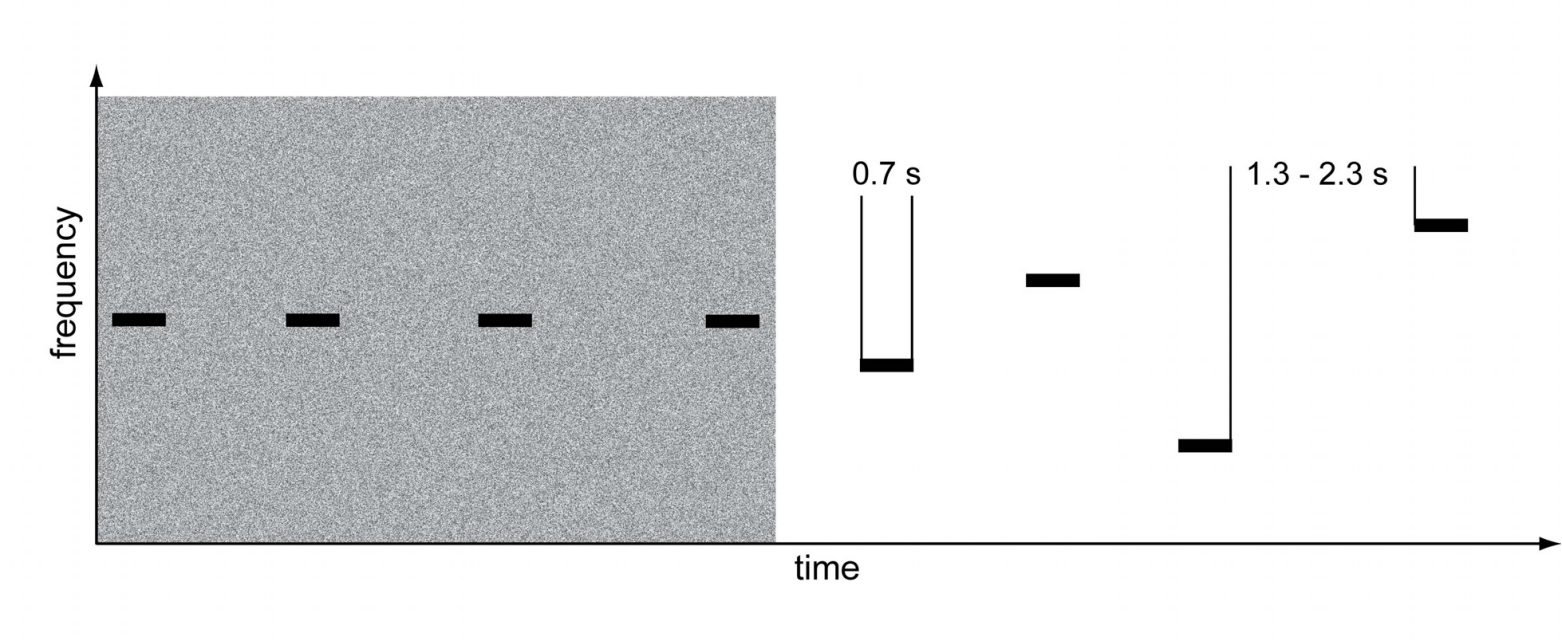
**Stimuli were presented in blocks of constant or random tone sequences**. All of those tone sequences were either presented in silence or in noise. The length of the tone stimuli was 0.7 s, the ISI was randomized between 1.3 and 2.3 s. Exemplary sound files are attached to Additional file 1.

Amplitude-modulated tones (modulation frequency 40 Hz, modulation depth 100%) of eight different carrier frequencies (250, 450, 700, 1000, 1370, 1850, 2500, 3400 Hz) with a duration of 0.7 s were used as test stimuli. The stimuli were concatenated in groups of 20 items of either the same carrier frequency (constant condition) or mixed across all eight carrier frequencies (random sequencing). The stimuli were prepared using MATLAB (The MathWorks Inc.) and CoolEdit (Syntrillium). The inter-stimulus interval was randomized between 1.3 and 2.3 s. In two of three noise conditions, an 8 kHz low-pass filtered white noise was added to the stimulus blocks, preceding the first tonal stimulus by 1 s. The total root-mean-square of the noise power was either 10 dB above or of the same power as the stimuli. The noises had 0.05 s linear rise and decay ramps. Each run contained blocks of randomly chosen different noise levels, with blocks of constant and random sequencing alternating. Figure 1 exemplarily depicts in schematic manner a spectrogram of parts of a constant sequencing block presented with noise followed by a random sequencing block presented without noise (see also additional file 1). During stimulus presentation, subjects were watching a silent movie, and after each of the six runs questions regarding the content of the movie were asked. This ensured that attention had been directed to the visual domain and was therefore distracted away from the auditory modality. In each sequencing and noise condition 160 trials were presented, amounting to 960 trials in total.

We used Presentation (Neurobehavioral Systems, Albany, CA, United States) to control the timing of sound presentation, and SRM-212 electrostatic earphones (Stax, Saitama, Japan) to transduce sound stimuli. All sounds were delivered diotically through silicon tubes (length: 60 cm; inner diameter: 5 mm) and silicon earpieces adjusted to fit into each individual ear.

Before starting the MEG acquisition, each subject's hearing threshold for the 1000 Hz carrier frequency test stimulus (TS) was measured for each ear. During the MEG session, the stimuli were presented at an intensity of 40 dB above this individual sensation level.

### Data acquisition and analysis

The auditory evoked fields were measured with a whole-head 275 channels MEG system (Omega; CTF Systems, Coquitlam, British Columbia, Canada) in a magnetically shielded and acoustically silent room. Subjects were instructed not to move their head position during the MEG measurement and monitored by video camera. The MEG data was recorded with a sampling rate of 600 Hz. The magnetic fields evoked by TS were averaged selectively for each noise and sequencing condition (irrespective of the carrier frequency), starting 0.35 s prior to TS-onset, and ending 1 s after TS-onset. Epochs containing field changes larger than 3 pT were rejected as artefacts. The overall percentage of rejected trials was less than 10%, with no significant difference between conditions.

For the analysis of the N1m response, which is the major deflection of the slow auditory evoked field [11], the averaged evoked fields of all conditions were 30 Hz low-pass filtered, and the baseline was corrected relatively to a pre-stimulus interval of 0.3 s. Initially, the maximal N1m response was identified at the time point of maximal root-mean square value of the global field power around 0.1 s after TS-onset. The N1m source locations and orientations were estimated by an equivalent current dipole model (one dipole in each hemisphere) for each subject individually. A 0.01 s interval around the N1m peak in the grand-averaged data of all conditions of each subject was used to estimate the equivalent dipolar current sources. Source estimations with insufficient goodness-of-fit (smaller than 95%) were excluded from further analysis, reducing the number of subjects from 17 to 15 (8 females, age range 21-30 years old). The estimated source was fixed in its location and orientation for each hemisphere of each subject as a spatial filter [17] to calculate source strength for each noise condition and each stimulus sequencing condition ('constant sequencing' and 'random sequencing'), irrespective of the TS carrier frequency. The maximal source strength in each noise and sequencing condition in the time range between 0.09 and 0.3 s was used for further statistical analysis of the N1m. Unfortunately, the auditory steady state responses [18] suffered from the low signal-to-noise ratio, possibly due to the masking sounds and the different carrier frequency TS. Therefore the auditory steady state responses were not analysed in the present study.

The maximum source strengths and latencies of the N1m responses elicited by the TS for each condition were analysed separately via planned comparisons, post hoc tests and repeated-measures analysis of variance (ANOVA) using two factors: SEQUENCING (constant and random) and NOISE_LEVEL (no noise, +/0 dB, + 10 dB).

## Results

**Figure 2:**
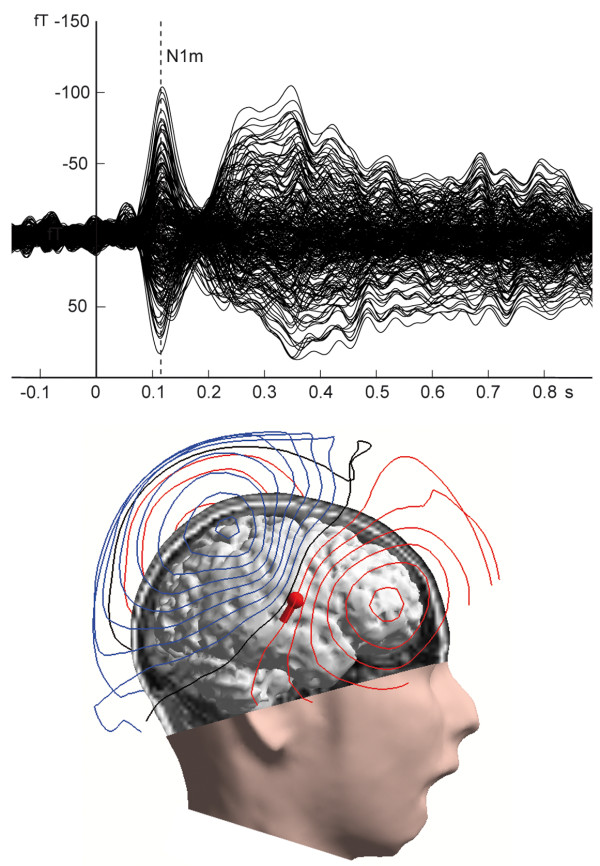
**Results of one representative subject**. Top: Averaged auditory evoked magnetic fields. The waveform exhibits a clear N1 m response peaking around the latency of 0.1 s. Bottom: Magnetic contour map and estimated single dipole at the latency of the maximal N1 m response are illustrated together with brain surface reconstructed from the individual MRI. Red and blue contour lines represent outbound and inbound magnetic field flow from and into the brain. The sphere and barrel in the brain indicate the location and orientation of a single equivalent current dipole in the right hemisphere.

**Figure 3:**
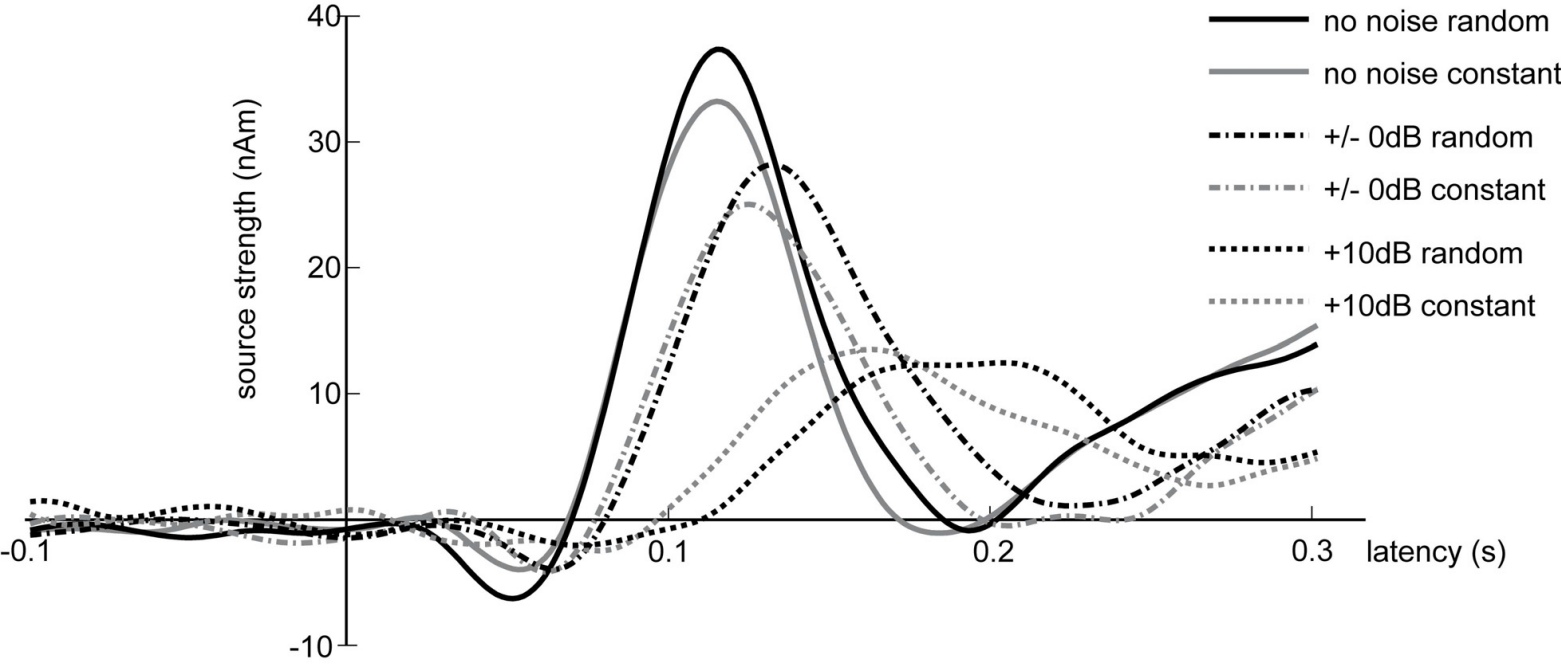
**Mean of source strength waveforms across all subjects (N = 15)**. Grey lines stand for the constant sequencing condition, and black lines stand for the random sequencing condition. Solid lines represent the no noise condition, dashed-dotted lines the +/0 dB condition and dotted lines the +10 dB noise condition.

**Figure 4:**
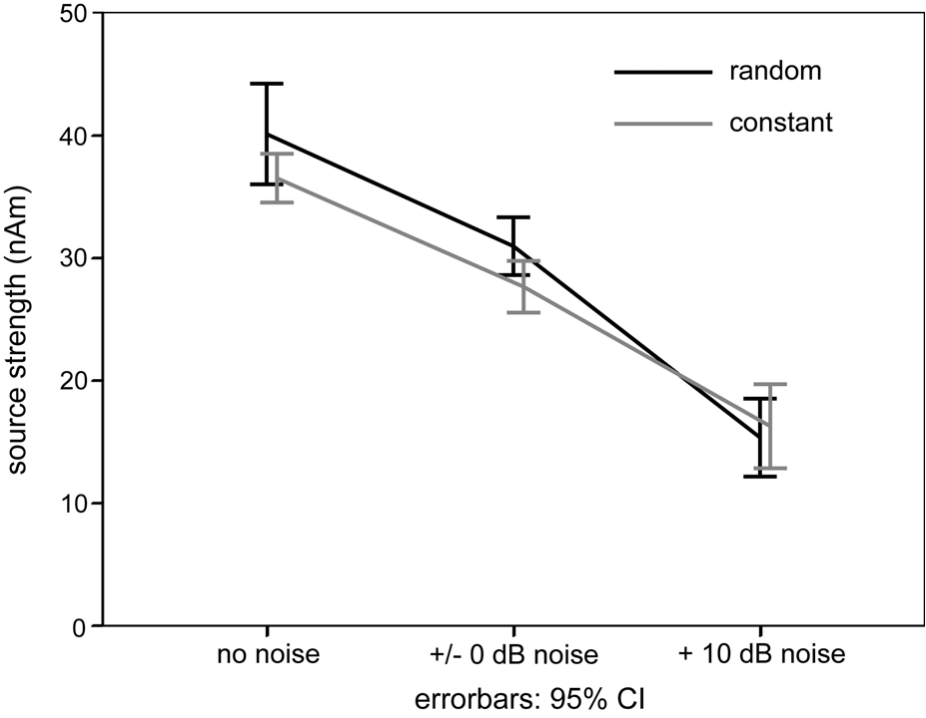
**Group means (N = 15) of the N1 m source strengths for each experimental condition**. The error-bars denote the 95% confidence interval limits of the arithmetic mean across subjects. The grey line marks the N1 m responses elicited by the test stimulus during the constant sequencing, and the black line marks the N1 m responses during the random sequencing.

Auditory evoked fields, corresponding contour map, as well as the estimated source location of the N1m response in the right hemisphere overlaid on a brain reconstructed from individual magnetic resonance images of one representative subject are displayed in Figure 2. A distinct N1m response peaking around 0.1 s is discernible. The contour map shows a clear dipolar pattern above the auditory cortex. Figure 3 depicts the mean source waveform for each condition averaged across all subjects (for individual source waveforms see the additional file 2). When presented in silence (no noise condition), the tones elicited a larger N1m source strength during random sequencing than during constant sequencing. With increasing noise level, the difference between random and constant sequencing decreased, and vanished completely in the condition when the noise intensity exceeded the stimulus intensity by 10 dB (Figure 4). A Kolmogorov-Smirnov test did not indicate significant deviation from normal distributions for both, source strength and latencies (see additional file 3). Mauchleys test indicated that the assumption of sphericity had been violated for source strength and latency. Therefore the reported values of the ANOVAS are Greenhouse-Geisser corrected. The repeated-measures ANOVA evaluating N1m source strength showed a significant main effect for NOISE_LEVEL (F(1.20,16.84) = 70.86 p < 0.001), and a significant interaction between SEQUENCING and NOISE_LEVEL (F(1.56,21.88) = 4.65, p < 0.05). Planned comparisons showed significantly larger source strengths for random compared to constant sequencing in the no noise condition (t(14) = -2.1, p < 0.05 (one-tailed)). Post hoc tests (Bonferroni test, df = 28, MSQe = 10.619) did not reveal any significant differences between constant and random sequencing for the +/-0 dB NOISE_LEVEL (p = 0.124) condition nor for the +10 dB NOISE_LEVEL (p = 1.0) condition.

**Figure 5:**
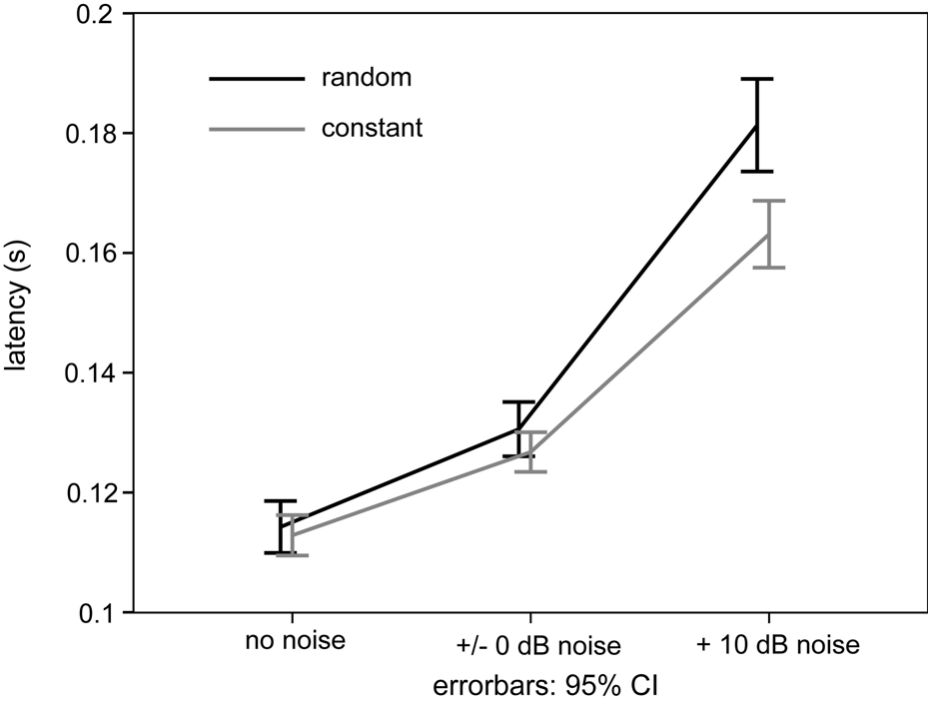
**Group means (N = 15) of the N1 m latencies for each experimental condition**. The error-bars denote the 95% confidence interval limits of the arithmetic mean across subjects. Grey line: N1 m responses elicited by the test stimulus during the constant sequencing; black line: N1 m responses during the random sequencing.

The N1m latency increased with increasing noise level. The largest difference between constant and random sequencing was found in the +10 dB noise condition (Figure 5). The repeated-measures ANOVA for N1m latency showed significant main effects for NOISE_LEVEL (F(1.34,18.8) = 142.869, p < 0.001) and SEQUENCING (F(1,14) = 25.447, p < 0.001), and a significant interaction between SEQUENCING and NOISE_LEVEL (F(1.39,19.52) = 16.74, p < 0.001). Post hoc tests (Bonferroni, df = 28, MSQe = 32.144) revealed a significant difference in latency between sequencing conditions for the +10 dB NOISE_LEVEL (p < 0.001), but not for the +/-0 dB NOISE_LEVEL (p = 1.0) nor for the no noise condition (p = 1.0). Thus, source strength values in the two different sequencing conditions converge with increasing noise level, while peak latencies diverge such that latencies for randomly presented TS increase stronger than those presented in a constant sequencing manner.

## Discussion

In the present study, subjects were exposed either to test stimuli (TS) presented in silence, or to TS embedded in broad-band noise. We found that the N1m source strength decreased with increasing noise level, as previously reported [19]. Additionally, we found that the noise level had different impacts on the constant sequencing and the random sequencing conditions. In the no noise condition, the source strength of the N1m evoked component was larger during random sequencing than during constant sequencing. However, this pattern changed when noise was added, yielding an interaction between the factors SEQUENCING and NOISE_LEVEL. This interaction shows that the processing in the auditory path and ultimately in the auditory cortex was differentially influenced by the acoustic environment depending on the different sequencing conditions.

The significant interaction between SEQUENCING and NOISE_LEVEL may arise from masking effects of simultaneously presented broad-band noise on the neural activity corresponding to the TS. In the silent surrounding, the TS presented in constant sequencing elicited smaller N1m responses compared to random sequencing because the identical neural population was repeatedly activated and therefore habituated. In the noisy surrounding, however, all auditory neurons were constantly stimulated by the broad-band noise - regardless of the sequencing order of the TS. The noise thus might have "evened out" the habituation effect of the TS. The higher source strength for constant stimulation in noise that was reported in earlier studies [13,14] was probably caused by differential masking effects of the band-eliminated noises for constant and random sequencing.

While habituation might account for some of the interaction between SEQUENCING and NOISE_LEVEL regarding the N1m source strength, it alone cannot explain the latency differences between CONSTANT and RANDOM sequencing in the +10 dB noise conditions. In previous behavioural studies [20,21] it was shown that signal in noise detection is facilitated by cuing the frequency of the tone to be detected compared to no cuing. In those studies, cuing was most effective when cue and target were identical. Comparable results were also found by Okamoto and colleagues [13], who reported faster reaction times and lower error rates in a detection task using tone stimuli overlaid with band-eliminated noise presented in constant sequencing (i.e. cued) compared to randomly sequenced stimuli (i.e. not cued). Additionally, they found stronger N1m activity and shorter latencies for the constant condition than for the random condition. These results suggest that auditory focused attention can increase the amplitude and shorten the latency of the N1m cortical source corresponding to the task relevant auditory signal. In our study, however, the auditory stimuli were not only task irrelevant, but subjects even had to focus their attention to another, the visual domain. With the subjects' attention not directed to the auditory domain, similar effects have been shown using band-eliminated noises and tones [14]. In this MEG experiment, the frequency of the tonal stimulus was at the centre frequency of the eliminated frequency band, and the stimuli were presented either in a constant or in a random fashion. As in our present study, the constantly presented stimuli yielded shorter latencies. The latency difference between the constant and random sequencing conditions in the present study might reflect the amount of time needed to involuntarily allocate attentional resources to the neural population tuned to the TS carrier frequency. To explain our results regarding latency differences between sequencing conditions merely with mechanisms of cuing would not suffice though, because then one would as well expect shorter latencies in the no-noise condition. We attribute the results of our present experiment to involuntary bottom-up driven neural mechanisms involved in the automatic tracking of auditory sequences [14], and we consider our finding to be comparable to simultaneous sound segregation phenomena described in earlier studies. During constant sequencing in broad-band noise, the sequence of TS of the same frequency is clearly separated from the noisy background. In the random sequencing condition, the series of TS is not separated from the noise as clearly as for the constant sequenced stimuli. The series of stimuli with constant frequency could be tracked more easily and the single entities were detected earlier. In the no noise condition, however, this constant tracking was not necessary since detection of the TS was very easy for both, the constant and the random sequencing condition. This seems to be the reason why no significant latency difference between constant and random sequencing in the silent condition was observed.

## Conclusions

The present data demonstrate that besides well-known neural response declining phenomena such as refractoriness, stimulus specific adaptation, or habituation another mechanism, elicited by constant signal sequencing, plays an important role in auditory signal in noise processing under distracted attentional conditions. This bottom-up driven involuntary neural mechanism may appropriately and automatically adjust the distribution of processing resources based on the surrounding noise level, resulting in better auditory performance in noisy environments.

## Authors' contributions

LL and HO conceived of and designed the study, LL performed the data collection and the data analysis. LL drafted the manuscript. HO, HS and CP improved the manuscript. All authors discussed the results and approved the final version of the manuscript.

## Supplementary Material

Additional file 1 **Schematic spectrograms and audible exemplary portions of stimuli blocks used**. Click here for file

Additional file 2 **Individual source waveforms for the +10 dB conditions**. Click here for file

Additional file 3 **Result of the Kolmogorov-Smirnov tests for latency and source strength**. Click here for file
