# Supplementary material for: Bottom-up driven involuntary attention modulates auditory signal in noise processing
Source: BMC Neurosci. 2010 Dec 30;11:156. doi: 10.1186/1471-2202-11-156 (PMC3022880; doi:10.1186/1471-2202-11-156)

Supplemental material: exemplary sound files

Constant sequencing, no noise:

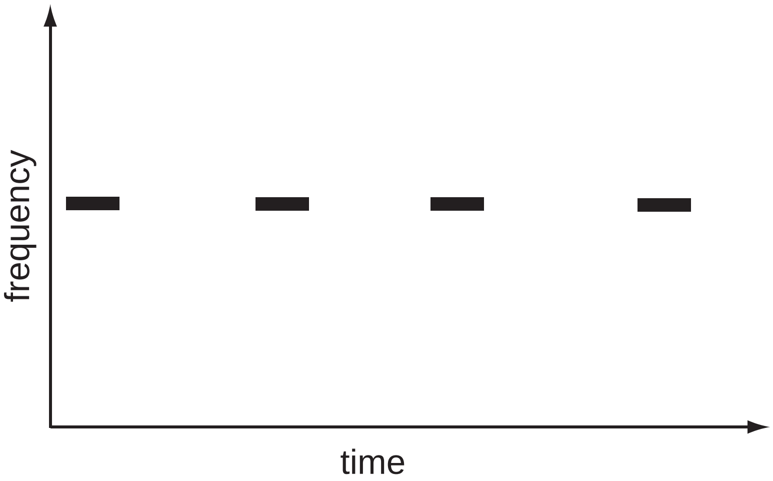

Random sequencing, no noise:

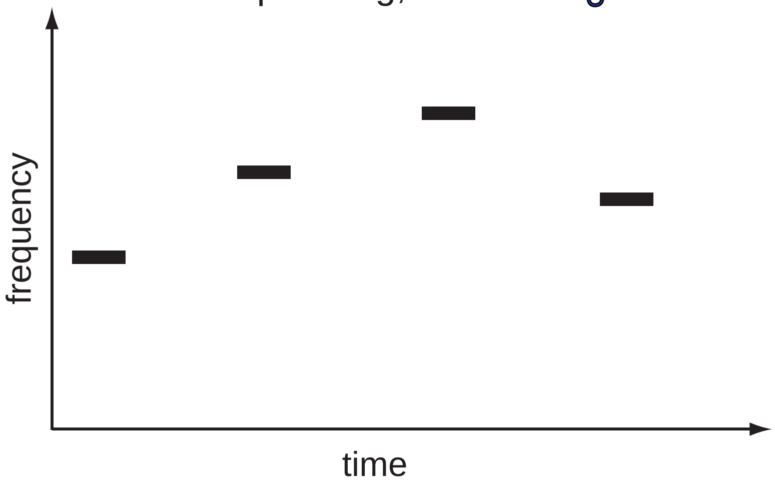

Constant sequencing, +/- 0dB noise:

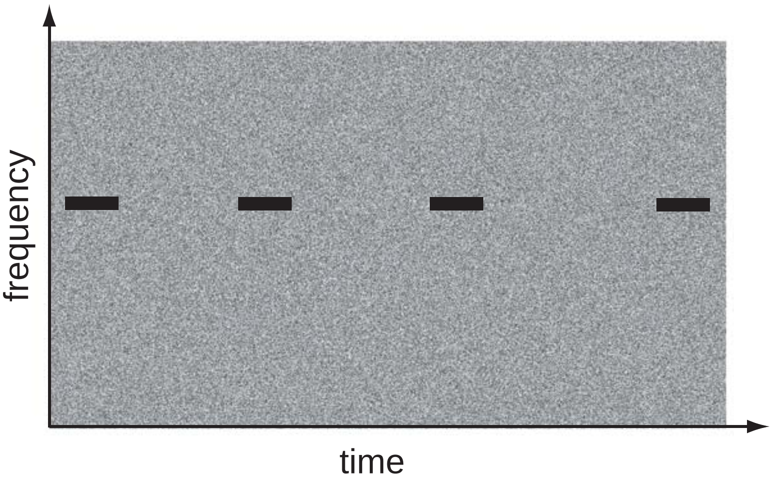

Random sequencing, +/- 0dB noise:

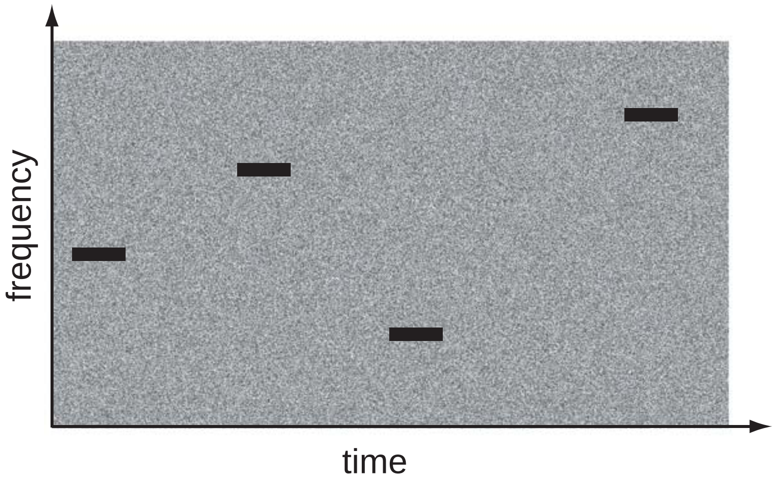

Constant sequencing, +10dB noise:

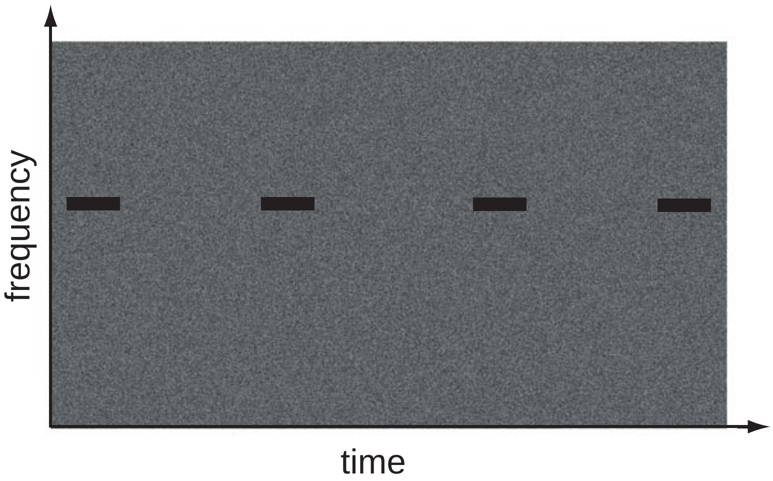

Random sequencing, +10dB noise:

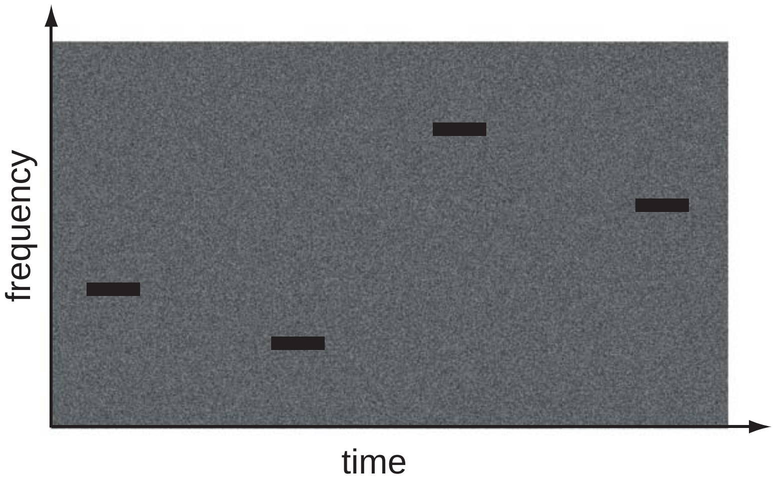

Supplement: Additional file 1 — Schematic spectrograms and audible exemplary portions of stimuli blocks used. [file 1471-2202-11-156-S1.PDF]
