# Supplementary material for: Bottom-up driven involuntary attention modulates auditory signal in noise processing
Source: BMC Neurosci. 2010 Dec 30;11:156. doi: 10.1186/1471-2202-11-156 (PMC3022880; doi:10.1186/1471-2202-11-156)

Individual source waveforms for the +10 dB conditions

Each row represents the data of one subject. The left column shows the source wave forms of the left hemisphere, the right column depicts the source wave forms of the right hemisphere. The scaling is consistent for each subject. The time window we used for determining the N1m peak ranged from 0.09 to 0.3 s. In cases where there was some ambiguity, we determined the peak by calculating the mean latency for the condition and hemisphere from subjects with clear peaks, and then selected the peak closest to this mean latency. These cases are marked.

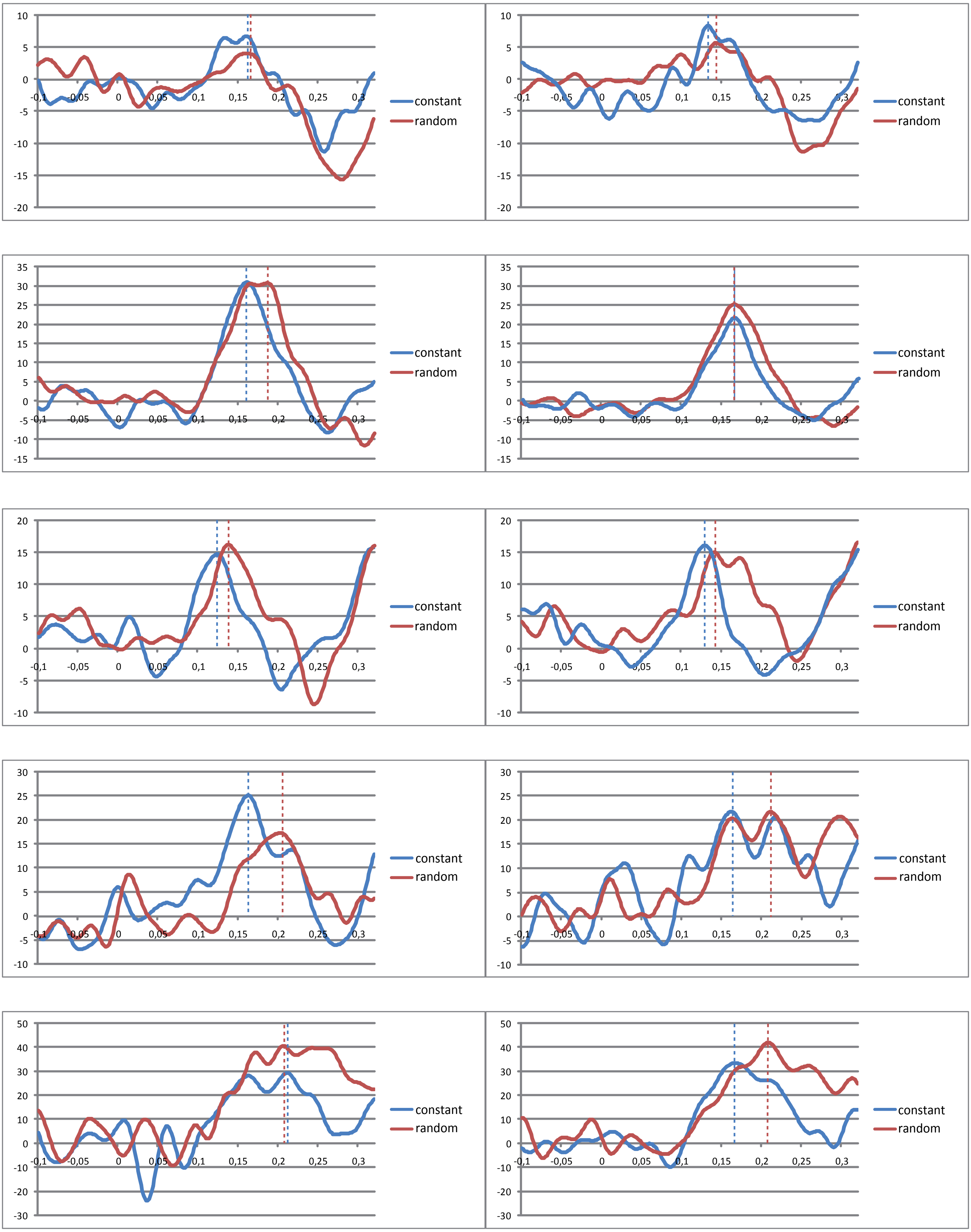

random: average method

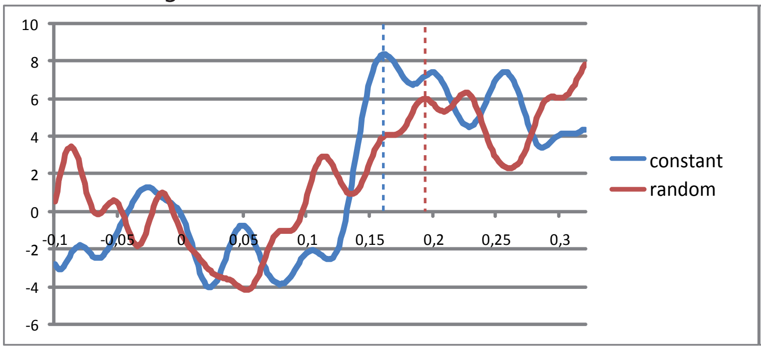

constant and random: average method

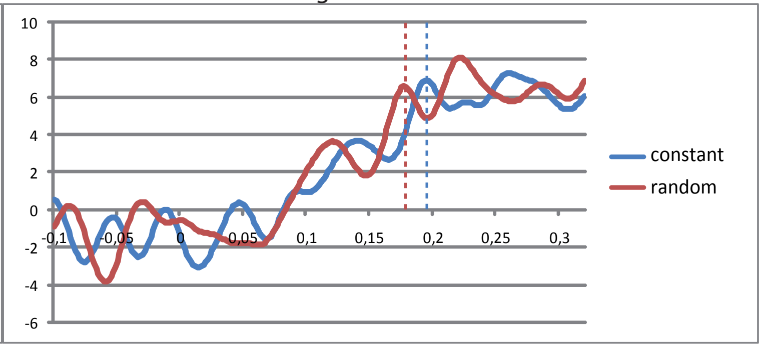

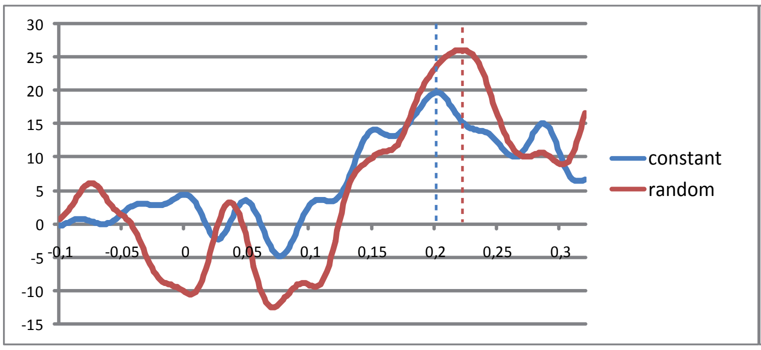

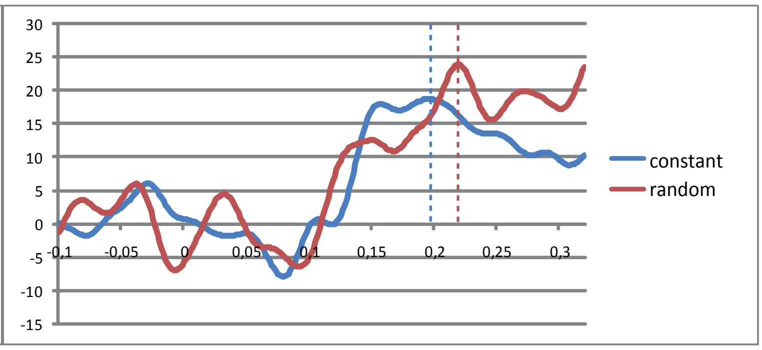

random: average method

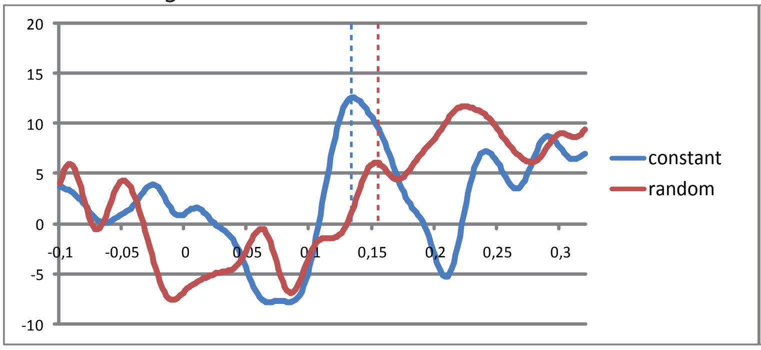

random: average method

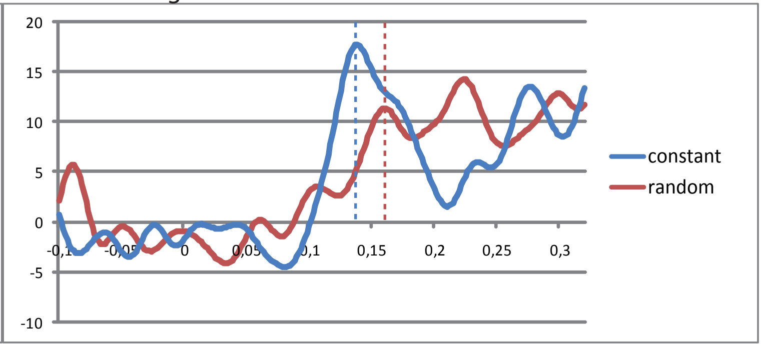

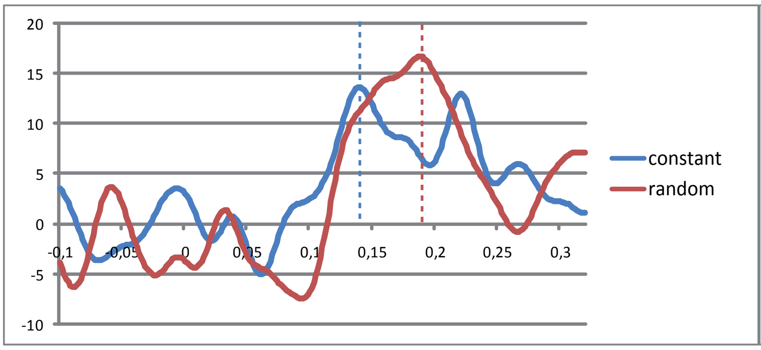

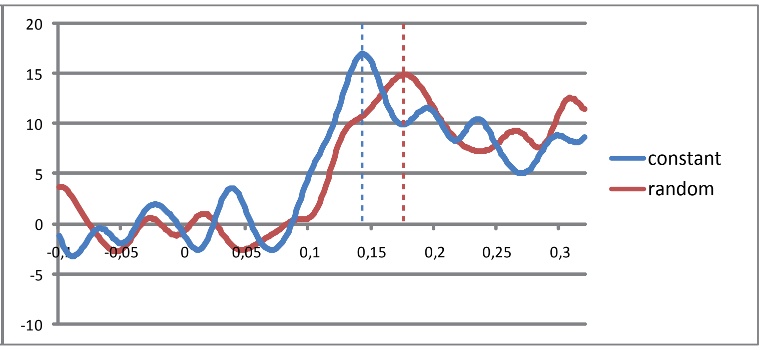

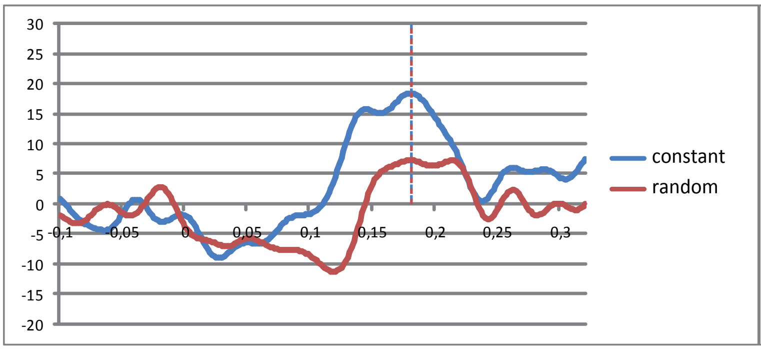

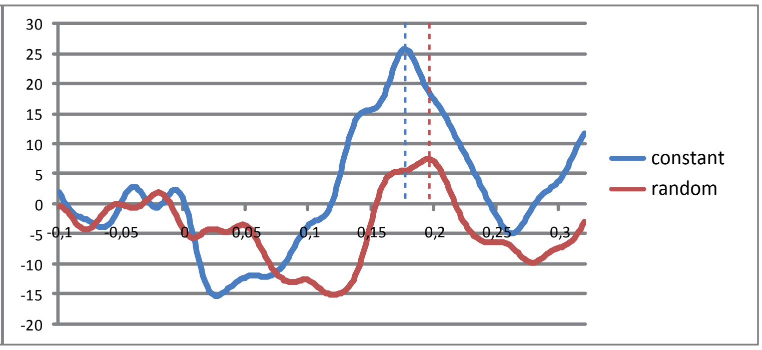

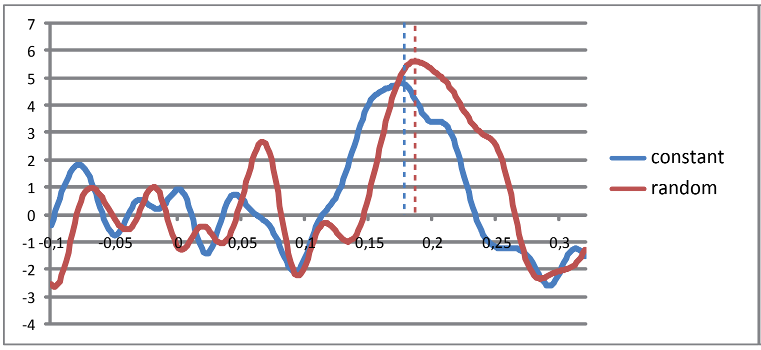

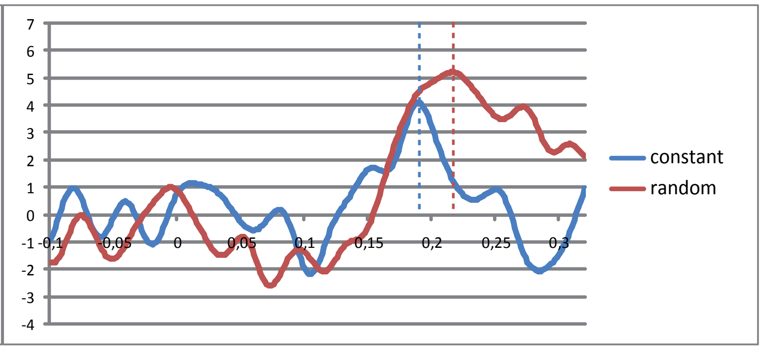

random: average method

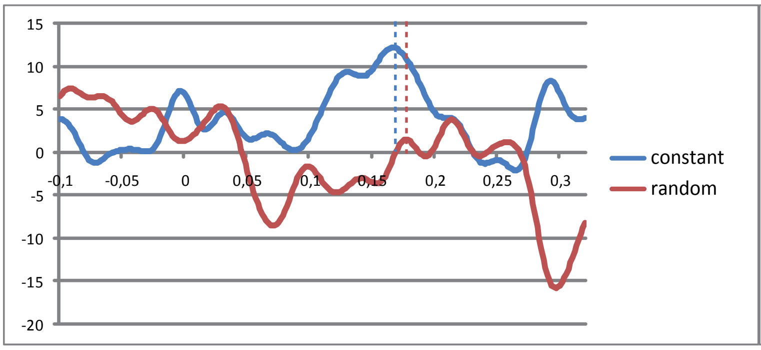

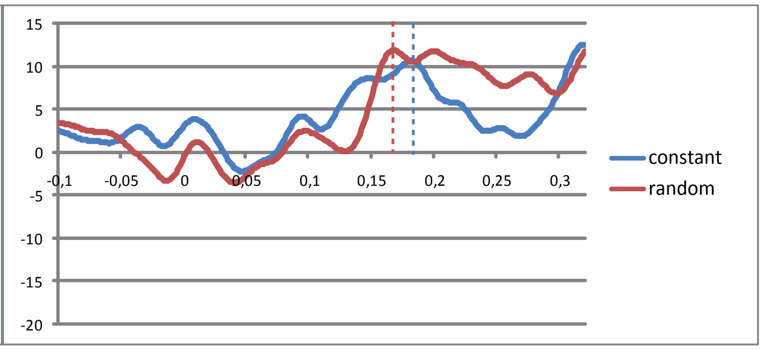

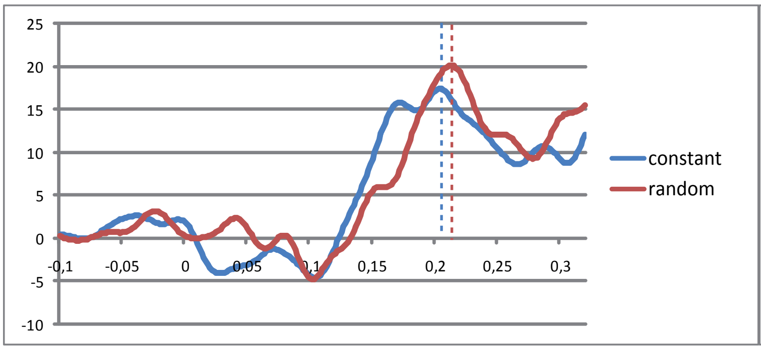

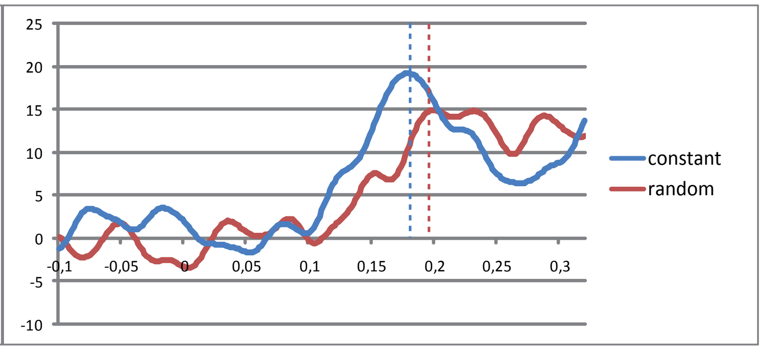

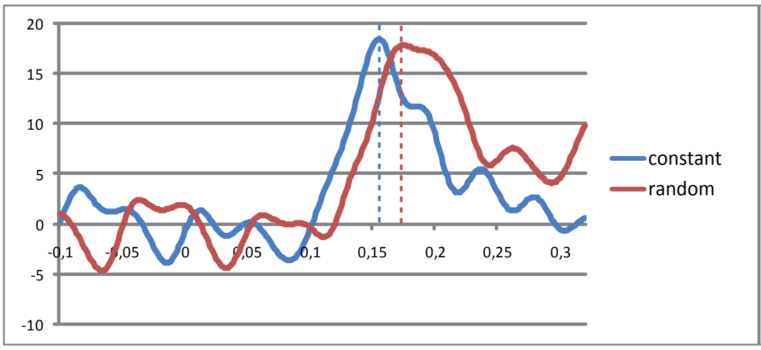

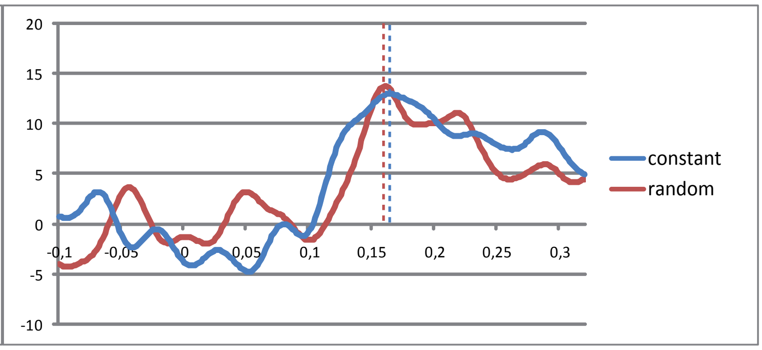

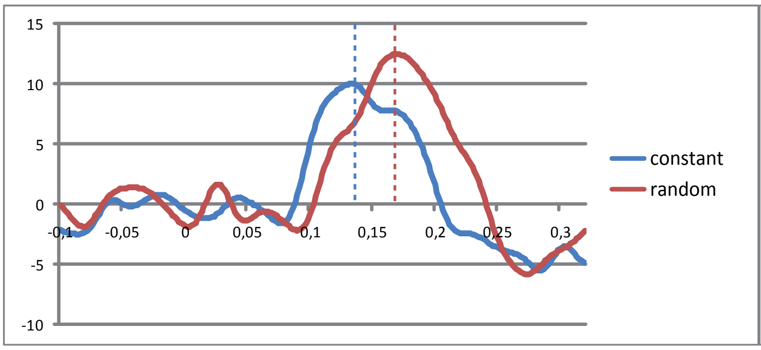

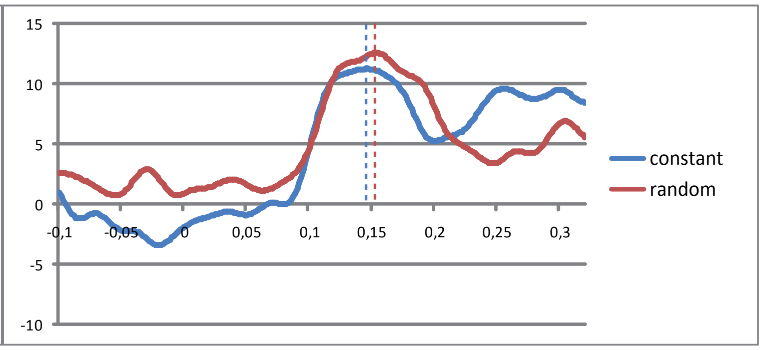

Supplement: Additional file 2 — Individual source waveforms for the +10 dB conditions. [file 1471-2202-11-156-S2.PDF]
