# Supplementary material for: Bottom-up driven involuntary attention modulates auditory signal in noise processing
Source: BMC Neurosci. 2010 Dec 30;11:156. doi: 10.1186/1471-2202-11-156 (PMC3022880; doi:10.1186/1471-2202-11-156)
Supplement: Additional file 3 — Result of the Kolmogorov-Smirnov tests for latency and source strength. [file 1471-2202-11-156-S3.PDF]

Kolmogorov-Smirnov test for latency

Condition	Statistic	df	Significance
no noise constant	0.199	15	0.114
+/-0dB constant	0.161	15	0.2
+10dB constant	0.163	15	0.2
no noise random	0.171	15	0.2
+/-0dB random	0.18	15	0.2
+10dB random	0.129	15	0.2

Kolmogorov-Smirnov test for source strength

Condition	Statistic	df	Significance
no noise constant	0.141	15	0.2
+/-0dB constant	0.171	15	0.2
+10dB constant	0.127	15	0.2
no noise random	0.149	15	0.2
+/-0dB random	0.145	15	0.2
+10dB random	0.15	15	0.2
